# Dual-Stream Attention-Enhanced Memory Networks for Video Anomaly Detection

**DOI:** 10.3390/s25175496

**Published:** 2025-09-04

**Authors:** Weishan Gao, Xiaoyin Wang, Ye Wang, Xiaochuan Jing

**Affiliations:** 1 China Aerospace Academy of Systems Science and Engineering, Beijing 100048, China; gaowsh518@163.com (W.G.); wxy690@126.com (X.W.); 15066241262@163.com (Y.W.); 2 Aerospace Hongka Intelligent Technology (Beijing) Co., Ltd., Beijing 100048, China

**Keywords:** video anomaly detection, deep learning, video understanding, attention mechanism

## Abstract

Weakly supervised video anomaly detection (WSVAD) aims to identify unusual events using only video-level labels. However, current methods face several key challenges, including ineffective modelling of complex temporal dependencies, indistinct feature boundaries between visually similar normal and abnormal events, and high false alarm rates caused by an inability to distinguish salient events from complex background noise. This paper proposes a novel method that systematically enhances feature representation and discrimination to address these challenges. The proposed method first builds robust temporal representations by employing a hierarchical multi-scale temporal encoder and a position-aware global relation network to capture both local and long-range dependencies. The core of this method is the dual-stream attention-enhanced memory network, which achieves precise discrimination by learning distinct normal and abnormal patterns via dual memory banks, while utilising bidirectional spatial attention to mitigate background noise and focus on salient events before memory querying. The models underwent a comprehensive evaluation utilising solely RGB features on two demanding public datasets, UCF-Crime and XD-Violence. The experimental findings indicate that the proposed method attains state-of-the-art performance, achieving 87.43% AUC on UCF-Crime and 85.51% AP on XD-Violence. This result demonstrates that the proposed “attention-guided prototype matching” paradigm effectively resolves the aforementioned challenges, enabling robust and precise anomaly detection.

## 1. Introduction

As urban security demands escalate, video anomaly detection (VAD) is increasingly implemented in intelligent surveillance systems to identify anomalous events in lengthy unedited videos, enhancing system response efficiency while minimising manual surveillance costs [1]. Current VAD approaches are primarily categorised into four types based on annotation granularity: fully supervised, semi-supervised, unsupervised, and weakly supervised [2]. While fully supervised techniques can theoretically attain superior accuracy, their dependence on precise frame-level annotations incurs significantly high labour costs. It limits the capacity of the model to generalise across varied real-world situations. Conversely, weakly supervised methods necessitate merely video-level annotations, achieving a commendable equilibrium between labelling efficiency and detection efficacy, so positioning them as a pivotal technological avenue for addressing extensive real-world surveillance challenges.

Despite its advantages, the prevailing weakly supervised video anomaly detection (WSVAD) paradigm, which typically employs a multi-instance learning (MIL) framework [3], faces significant challenges that this paper aims to address. These challenges can be categorised into three main areas: high false alarm rates, indistinct feature boundaries stemming from the MIL framework itself, and critical limitations in temporal modelling.

Weakly supervised video anomaly detection (WSVAD) typically employs a multi-instance learning (MIL) framework [3], wherein the video is regarded as a “bag” comprising many segments, hence converting the anomaly detection problem into the identification of “key instances” within the bag. This technique inherently causes the model to concentrate on the highest-scoring anomalous segments, therefore inadequately modelling the numerous normal segments. This inclination to prioritise the abnormal over the normal results in two significant challenges: firstly, in the spatial dimension, the model fails to comprehend normal patterns fully and is susceptible to misclassifying normal movements in intricate contexts as abnormal, such as detecting a sudden change in illumination during nighttime surveillance or a brief presence in a crowd as a false alarm. Secondly, in the feature dimension, the model struggles to establish a distinct decision boundary, causing similar motion characteristics, such as normal walking and abnormal running, to be easily conflated, which results in feature redundancy and diminished discriminative capacity.

Moreover, current methodologies depend on single-scale convolution or attention mechanisms for temporal modelling, hindering the ability to capture both the immediate onset of events and the long-term progression of the process [4,5]. This is susceptible to feature deterioration in deep networks. To address the aforementioned challenges, this paper offers a dual-stream attention and memory-enhanced network that fundamentally tackles the issue of boundary blurring. To specifically tackle the temporal modelling limitations, this study also develops a hierarchical multi-scale temporal encoder architecture. It ensures seamless information flow via residual connections, leveraging the robust capabilities of temporal convolutional networks [6] and multi-scale kernels to effectively capture intricate dependencies across various time scales. The core of the proposed method, the dual-stream attention module, employs a dual-channel attention method to assign weights to features in both spatial and channel dimensions, therefore precisely identifying essential regions in the video and mitigating irrelevant information interference. By individually generating normal and abnormal memory banks, the model may independently learn the prototype distributions of both sample types, in contrast to prior memory networks that solely rebuilt the normal pattern [7,8]. The integration of the memory improvement mechanism with the Top-K selection process aligns and refines the features, leading to highly discriminative feature representations and significantly mitigating the probability of misjudgement. This primary research focus and contributions of this paper are outlined as follows:

- A hierarchical multi-scale temporal encoder architecture is developed, incorporating multi-scale modelling capabilities and integrating residual connectivity to increase the representation of intricate temporal aspects.
- A dual-stream attention-enhanced memory network is introduced. This module utilises a bidirectional attention mechanism to refine features before interacting with the memory banks, enabling independent modelling of the prototype distributions of normal and abnormal samples. This design enables the model to effectively differentiate between the two feature types under weakly supervised settings.
- The suggested method, thoroughly tested on two public datasets, UCF-Crime and XD-Violence, surpasses most contemporary popular methods while maintaining RGB unimodal inputs. It exhibits robust generalisation capability and practical application possibilities.

## 2. Related Works

### 2.1. Weakly Supervised Video Anomaly Detection

While our work focuses on weakly supervised learning, another prominent paradigm is unsupervised learning, where models are trained without any video-level labels. Many of these methods focus on generating pseudo-supervision to guide the training. For instance, C2FPL [9] creates coarse-to-fine pseudo-labels via clustering, DvAnNet [10] refines pseudo anomaly scores using a dual-branch network, and GCL [11] employs a generative cooperative learning framework for cross-supervision. Other recent approaches, like CLAP [12], leverage large-scale cross-modal pre-trained models for this task. In contrast, weakly supervised video anomaly detection has evolved along two primary technical avenues: single-stage approaches grounded in multiple instance learning (MIL) and two-stage methodologies that employ pseudo-labelling for self-directed training.

The single-stage methodology is exemplified by the fundamental MIL framework introduced by Sultani et al. [3], which categorises movies into positive and negative “packets” and enhances the separation between the highest scoring segments in these packets using sorting loss. Nonetheless, the primary issue with this methodology is its excessive dependence on the presumption that “anomalous clips possess pronounced features,” with its optimisation objective centred on “identifying” anomalies, significantly overlooking the modelling of extensive and varied normal patterns. Subsequent works, including the top-k mechanism by Tian et al. [13] and the centre-of-mass loss proposed by Wan et al. [14], aim to enhance feature extraction; however, they remain anchored to this fundamental concept. Another approach, BN-WVAD [15], is inspired by the statistical insight that features of abnormal events often exhibit outlier characteristics. It leverages the Divergence of Feature from the Mean (DFM) from BatchNorm statistics as an additional, noise-resilient anomaly score to amend the predictions of the primary classifier. Consequently, these methods encounter difficulties in managing false alarms and generalising the feature space, complicating the distinction between normal behaviours in intricate contexts and genuine anomalies.

Another technical approach is to employ pseudo-labels for self-supervised training. For instance, Feng et al. [16] and Zhang et al. [17] improve the quality of pseudo-labels via generators or evaluation methods. Similarly, OE-CTST [18] develops a temporal transformer framework that uses an Outlier Embedder and a Cross Temporal Scale Transformer to better model the temporal dynamics of both long and short anomalies. While these methods enhance the precision of anomaly detection, their pseudo-labels are predominantly derived from the higher-scoring suspected anomalous segments, neglecting the significance of structural information from normal samples, thereby leaving the distinction between normal and anomalous ambiguous. Furthermore, specific approaches, like the graph convolutional network proposed by Li et al. [19], attempt to simulate fragment interactions; nevertheless, their static topology poses challenges in adapting to the dynamically evolving temporal dependencies. In contrast, MTFL [20] proposes a Multi-Timescale Feature Learning method, which enhances feature representation by employing a Video Swin Transformer on short, medium, and long temporal tubelets. The approaches above typically exhibit insufficient residual connectivity in the construction of deep networks, resulting in feature degradation issues and constraining the deep expressive capability of the model.

In summary, the design of contemporary mainstream technological approaches presents several intrinsic limitations, particularly an inability to precisely target critical spatio-temporal information. To address this, various attention mechanisms have been explored. Some approaches generate attention maps using a hybrid of classic and deep learning methods. For instance, Shoaib et al. [21] proposes a visual attention mechanism where motion regions are first identified using a background subtraction algorithm before being processed by a 3D CNN. While effective, such methods can be sensitive to complex background dynamics. More recent works have focused on end-to-end learnable attention, often in multi-modal settings. A prominent example is the work by Ghadiya et al. [22], which introduces a hyperbolic Lorentzian graph attention to capture hierarchical relationships between audio-visual features. Recent trends also leverage large pre-trained models from the language domain to enhance video understanding. AnomalyCLIP [4], for example, was the first to combine a Vision-Language Model (VLM) like CLIP with the MIL framework, using text prompts about normalcy to learn text-driven feature space directions for identifying anomalies. Similarly, MIL-BERT [23] adapts the BERT architecture to improve performance via explicit whole-video classification, where it aggregates all snippet features into a single video-level representation.

To resolve these problems in a unimodal setting, this paper develops a dual-stream attention and memory-enhanced network that directly addresses the issues of feature space border ambiguity and elevated false alarm rates via its dual-attention mechanism and separate normal and abnormal prototype memory banks. The hierarchical multi-scale temporal encoder we developed effectively addresses the limitations of temporal modelling and feature degradation through its multi-scale architecture and residual connections.

### 2.2. Memory Networks

Memory networks are neural networks equipped with external storage that can read and write data, capture long-term dependencies during training, and employ stored memory components to produce outcomes during inference. Memory networks were initially utilised for text-based question–answer activities to preserve long-term memory by storing memory items related to scene information. This type of model requires supervision at each layer during training, complicating the backpropagation training process. Sukhbaatar et al. [24] presented a continuous memory network that may be taught end-to-end to address this issue, hence broadening its applicability to other tasks.

At present, memory networks are primarily utilised for visual anomaly detection in unsupervised learning contexts. Gong et al. [7] introduced a memory-enhanced self-encoder to address the issue of excessive reconstruction of anomalous events by the self-encoder. Rather than directly supplying the input image to the decoder, the model interprets it as a query, collects the most pertinent memory elements from the memory module for integration, and subsequently, the decoder finalises the image reconstruction. Park et al. [25] contended that the conventional singular prototypical feature fails to encompass the multimodal attributes of normal data. Consequently, they introduced a novel memory module wherein each memory item embodies a normal modal prototypical feature, regulating the relationship between features and memory items through feature compactness loss and feature separation loss. Nevertheless, the technique continues to inadequately address the alignment issue between memory content and temporal structure, rendering its modelling of extended time-series events somewhat flawed.

Nonetheless, current memory network-based methodologies have some significant drawbacks. Firstly, a solitary memory module struggles to encapsulate the intricate dynamic information included in a video accurately. Secondly, the absence of precise labelling at the frame level hinders present approaches from effectively capturing the category information of video frames, hence constraining their applicability in weakly supervised learning contexts. Moreover, the majority of approaches employ a predetermined quantity of memory terms, a hyperparameter set manually that remains invariant throughout training, hence constraining the adaptability of the model to varying video contexts. To address the issues above, new research has commenced experimentation with the concept of integrating dual memory modules with uncertainty modelling to enhance adaptability to weakly supervised tasks; for instance, the DMU technique [26] exemplifies this approach. This work presents a memory information distillation module that integrates dual memory modules and dual attention techniques for both channel and spatial dimensions. The essence of the module is to adaptively discern “what” and “where” through the synergistic interplay of channel and spatial attention, hence extracting more distinctive traits. Subsequently, these enhanced capabilities are retained and disseminated in standard and atypical prototypes utilising the engineered split dual memory unit. This approach enables the model to reliably differentiate between normal and abnormal patterns and successfully adapt to poorly supervised environments, hence greatly enhancing detection performance and minimising false alarm rates.

## 3. Proposed Method

**Figure 1:**
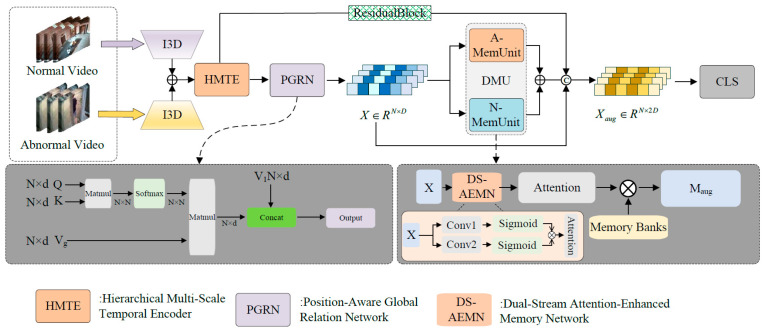
The overall architecture of the proposed method.

This paper proposes a weakly supervised video anomaly detection method designed to systematically tackle the fundamental problems of existing methods in temporal modelling and precise classification using a problem-driven, layer-by-layer architecture, as illustrated in Figure 1.

This paper firstly confronts the primary problem in the weakly supervised context: the proficient modelling of intricate temporal relationships. To tackle this difficulty, we developed the Hierarchical Multi-Scale Temporal Encoder (HMTE) to effectively capture intricate local and global dynamic properties via its multi-scale convolutional architecture (see Section 3.1 for further details).

Nonetheless, it is challenging to capture non-adjacent yet semantically linked global connections just by depending on the fixed receptive fields of HMTE. Consequently, we present the Position-Aware Global Relation Modelling module (PGRN) to augment the global contextual comprehension of the model by integrating the self-attention mechanism of content and locational data (see Section 3.3 for further details).

Upon acquiring robust spatio-temporal features, the primary objective is to execute precise and dependable anomaly differentiation. This is where the Dual-Stream Attention-Enhanced Memory Network (DEMN), the principal breakthrough of this study, is introduced. This module transitions from feature characterisation to prototype-level differentiation by creating a prototype memory and integrating it with bi-directional attention (see Section 3.2 for specifics).

The comprehensive architecture is supported by a dynamic residual module for deep structure (refer to Section 3.1 for specifics). It is concurrently trained using a multi-objective co-optimisation loss function (see Section 3.4 for further details).

### 3.1. Hierarchical Multi-Scale Temporal Encoder

**Figure 2:**
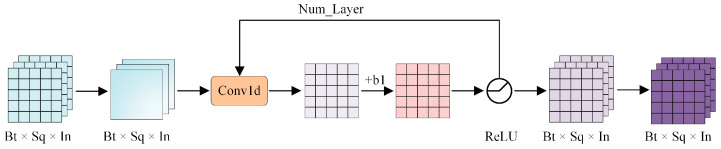
Hierarchical multi-scale temporal encoder architecture.

To address the challenge that conventional MIL frameworks struggle to adequately capture intricate temporal patterns and long-range dependencies, this paper develops a Hierarchical Multi-Scale Temporal Encoder (HMTE). The core role of the HMTE is to thoroughly extract both local temporal features and long temporal dependencies from video frame sequences. It achieves this through a multi-layer architecture of stacked dilated convolutions, as illustrated in Figure 2, allowing the model to perceive temporal dynamics across various scales.

The embedding layer employs hierarchical temporal feature extraction through a multilayer convolutional architecture, wherein each layer transforms the input sequence into a high-dimensional feature space via convolutional operations to elucidate the intricate dependency patterns of the time series. Let the input tensor be denoted as $X\in R^{B\times T\times D}$, with B representing the batch size, T indicating the time step duration, and D signifying the number of features per time step. The objective is to transform the input into an output tensor $Y\in R^{B\times T\times O}$, where O denotes the output feature dimension, via the embedding layer. The output of layer l can be articulated as:

(1) $$Y^{\left(l\right)}=f\left(W^{\left(l\right)}{{*}_{d}Y}^{\left(l-1\right)}+b^{\left(l\right)}\right),$$

In the equation above, ${*}_{d}$ represents the dilation convolution operation, $f\left(\cdot \right)\;$ is the nonlinear activation function ReLU, and $W^{\left(l\right)}$ and $b^{\left(l\right)}$ describe the convolution kernel and the bias term of layer l, respectively. The dilation convolution is defined as follows:

(2) $$Y^{\left(l\right)}\left[t\right]=\sum_{k=0}^{K-1}W^{\left(l\right)}\left[k\right]\cdot Y^{\left(l-1\right)}[t-r\cdot k],$$

K represents the dimension of the convolution kernel, whereas r denotes the dilation factor. Dilation convolution enhances the receptive field efficiently without augmenting the parameter count or the convolution kernel size, which is essential for managing extensive time series. By structuring distinct layers to utilise varying sequences of dilation factors (e.g., $r=(1,2,4,\ldots ,2^{L-1})$), the embedding layers can concentrate on diverse scales of temporal information across multiple layers, hence facilitating multi-scale feature extraction. The layer-by-layer integration of multilayer convolution and activation functions enables the model to extract both local and global time-dependent characteristics, thereby considerably improving its capacity to differentiate between complicated anomalous patterns and typical behavioural patterns.

**Figure 3:**
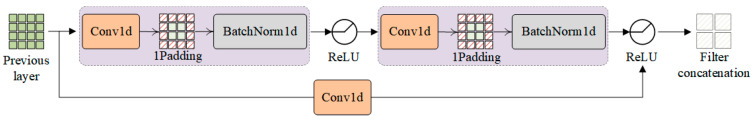
The dynamic residual feature enhancement module. This module is represented as the ‘ResidualBlock’ in the overall architecture in Figure 1.

To ensure that the HMTE can still be effectively trained after stacking multiple layers, this paper incorporates a dynamic residual feature enhancement module, as illustrated in Figure 3. The residual block is engineered to mitigate the vanishing gradient issue in deep networks by incorporating shortcut links, hence facilitating more efficient information flow over multiple layers. The fundamental procedure of the residual block commences with the input signal x. The input signal is initially processed by the first convolutional layer for feature extraction, which typically utilises a small convolutional kernel (e.g., 3 × 3) to collect local features. The convolutional layer alters the spatial configuration of the input signal into a collection of feature mappings, offering a more representative input for further processing. Upon completion of the convolution procedure, the feature mappings are transmitted to a batch normalisation layer. Batch normalisation aims to standardise the inputs of each layer by setting the mean of each feature to zero and the variance to one, hence mitigating internal covariate shift. This procedure not only accelerates convergence but also makes the model more stable. Following batch normalisation, the ReLU activation function is applied to the convolved and normalised feature mapping to incorporate nonlinear characteristics. The nonlinearly triggered feature mappings are subsequently input into a second convolutional layer for further feature extraction. Like the initial convolutional layer, the second convolutional layer utilises a 3 × 3 convolutional kernel and retains the same number of channels to preserve feature dimensionality. The output of this convolutional layer undergoes batch normalisation to help stabilise the learning of the model. At this juncture, the fundamental characteristics of the residual block are activated. Upon the completion of the second convolution and batch normalisation of the feature mapping, the output ${out}_{2}$ is summed with the original input x, constituting a crucial element in residual learning. This addition operation enables the model to preserve the original information of the input signal while also facilitating information movement within the deep network. It is articulated expressly as:

(3) $$Output=ReLU(\underset{Learning\;the\;residual\;function\;F(x)}{\underbrace{BN(Conv2(ReLU(BN(Conv1(x)))))}}+\underset{\mathrm{short}-\mathrm{circuit}\;\mathrm{connections}}{\underbrace{x}}),$$

### 3.2. Dual-Stream Attention-Enhanced Memory Network

After acquiring robust spatio-temporal features, the next critical challenge is to perform precise and reliable anomaly differentiation. To solve the problem that initial deep features often contain substantial duplicate and irrelevant information that obstructs final judgement, this paper introduces the Dual-Stream Attention-Enhanced Memory Network (DS-AEMN). The purpose of this module is twofold: first, its attention mechanism refines input features by focusing on essential spatio-temporal information; second, its learnable memory bank acts as prior knowledge to augment the features’ discriminative power.

The fundamental premise of the approach posits that anomalous events can be viewed as deviations from recognised typical patterns. To formalise this previous knowledge, two parallel, trainable memory banks are introduced: a normal memory bank ($M_{norm}\in R^{M\times D}$) and an abnormal memory bank ($M_{abn}\in R^{M\times D}$). Beyond simply improving accuracy, the goal of this dual-memory architecture is to make the model more transparent and interpretable. Each memory item, within these banks is intended to learn a semantically distinct pattern. For instance, different prototypes in $M_{norm}$ might learn to represent common normal events like ‘walking pedestrians’ or ‘passing cars’. Conversely, prototypes in $M_{abn}$ are expected to specialise in different types of anomalies, such as one prototype capturing ‘physical fights’ while another captures ‘sudden explosions’. This approach enables the model to statistically assess real-time inputs against these learned “standard” templates, allowing practitioners to gain insight into not just whether an anomaly occurred, but also what kind of event the model identified, thus establishing a definitive and transparent basis for discriminating.

Before comparing with the memorised templates, the purification of the input features is an essential step. Typically, a designated spatial area (“where did the event transpire?”) and a defined feature channel (“what type of event was it?”) are critical in a video clip. A bidirectional spatial attention module has been developed to identify and enhance this essential information dynamically. To attain this objective, the module separates the attention challenge into two basic components: localising physically significant locations (“where) and finding semantically significant channels (“what”). Spatial attention produces a spatial weight map using the convolution operation $A_{spatial}=\sigma ({Conv}_{1}(data))$, which emphasises the areas where events transpire. Simultaneously, channel attention allocates weights to each feature channel via $A_{channel}=\sigma ({Conv}_{2}(data))$, emphasising the most informative feature dimensions.

**Figure 4:**
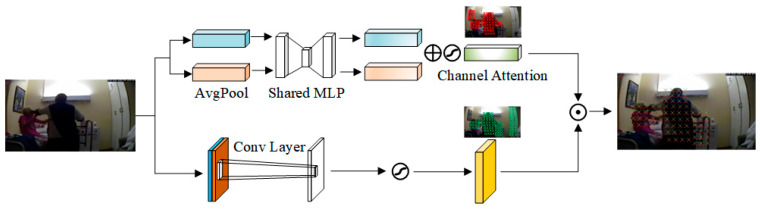
Detailed structure of the dual-stream attention component. This component is part of the DS-AEMN module shown in Figure 1.

The two attention modules do not function separately; instead, they combine synergistically through element-wise multiplication $A=A_{spatial}⊙A_{channel}$ to attain enhanced precision in concentration, as shown in Figure 4. The advantage of this fusion strategy is that it assigns a greater final attention weight to a feature only when it occupies a critical spatial location and simultaneously belongs to an essential feature channel. This synergy efficiently mitigates background noise and significantly enhances the signal-to-noise ratio of the feature. Ultimately, we utilise this aggregated attention weight on the input data to produce the improved feature $attention=\frac{A}{\sqrt{d}}\cdot data$, which establishes the groundwork for ensuing memory searches.

Figure 4 illustrates the structure of the dual-stream attention mechanism. The spatial attention module identifies the most informative spatial regions, while the channel attention module highlights the most discriminative feature channels. These two attention maps are then fused through element-wise multiplication to produce a refined attention weight map, which enhances the most relevant spatio-temporal features and suppresses irrelevant background noise. Upon acquiring the meticulously honed qualities, they are utilised as queries to engage with the memory bank. The Query process initially acquires a matrix of similarity scores between the input features and all memory templates by calculating the dot product and applying the softmax activation function $S=softmax\left(\frac{X\cdot M^{T}}{\sqrt{D}}\right)$, here $X\in R^{M\times D}$ represents the matrix of N input features, $M\in R^{C\times D}$ is the memory bank containing C prototype templates, and D is the dimension of the features. The softmax function is applied row-wise, converting the similarity scores for each input feature into a probability distribution over the C prototypes. This paper additionally utilises the Top-K selection technique to enhance the robustness of query results and mitigate the influence of secondary templates. This technique picks the K templates that most closely align with the input and calculates the final query score $S_{K}^{topK}=\frac{1}{K}\sum_{j=1}^{K}S_{i,j}$ by averaging their similarity scores, topK denotes the set of indices for the top K scores in that row. This final score, $S_{K}^{topK}$, consistently indicates the degree to which the input corresponds to an entire category of prototype patterns.

The advantages of the query process manifest in two aspects: the generation of a discriminant score and the acquisition of a memory enhancement feature, $M_{aug}=S\cdot M$. Here, $M_{aug}$ is a reconstructed feature representation for the input, created by computing a weighted sum of all prototype vectors from the memory bank (M), using the similarity scores in the matrix S as the corresponding weights. This enhancement feature efficiently addresses essential information that may be confusing or absent in the original representation by integrating the matched prototype information back into the input features. Specifically, for sparse anomalous occurrences, the input samples often engage just a limited number of templates in the anomaly memory bank, allowing the model to selectively employ the most pertinent prior knowledge for enhanced recognition of certain anomaly kinds.

### 3.3. Position-Aware Global Relation Network

While the HMTE (Section 3.1) adeptly captures local timing characteristics, its rigid convolutional architecture possesses an intrinsic constraint: it struggles to form dynamic, content-oriented global associations between non-adjacent yet semantically significant keyframes. To overcome this bottleneck, this paper introduces the Position-Aware Global Relation Network (PGRN). The primary role of the PGRN is to enhance the global contextual refinement of the feature sequences produced by HMTE. It achieves this by incorporating a modified self-attention mechanism, which successfully integrates content-driven similarity with temporal proximity by adding a relative distance-based attention bias to the conventional scaled dot-product attention.

Consequently, with the query (*Q*), key (*K*), and value (*V*) matrices, the resultant attentional weight matrix A is conclusive and the attentional $O_{Attn}$ may be succinctly expressed as:

(4) $$A=Softmax(\frac{QK^{T}}{\sqrt{d_{h}}}+B_{pos}),\;O_{Attn}=AV,\;$$

where $d_{h}$ represents the dimension of each attention head, and the bias term $B_{pos}$ is calculated from the relative distances $\left|i-j\right|$ via a compact learnable network, integrating essential location-sensing functionalities into the model.

The suggested location-aware self-attention layer is integrated into conventional Transformer encoder blocks within the overall design. Each encoder block comprises a multi-head self-attention mechanism and a feed-forward neural network (FFN), incorporating residual connections and layer normalisation to facilitate efficient information transfer and training stability. For layer l−1, the comprehensive computational procedure of layer l can be articulated as:

(5) $$X_{Attn}^{\prime }=\mathrm{LN}\left(X^{\left(l-1\right)}+\mathrm{MultiHeadAttn}\left(X^{\left(l-1\right)}\right)\right),\;X^{\left(l\right)}=\mathrm{LN}\left(X_{Attn}^{\prime }+\mathrm{FFN}\left(X_{Attn}^{\prime }\right)\right),\;$$

Thus, the PGRN module transcends fixed spatio-temporal neighbourhood restrictions, capturing genuine global dependencies that integrate content and location information, so facilitating a comprehensive understanding of the deeper semantics of the entire video sequence.

### 3.4. Multi-Objective Collaborative Optimisation Loss Function

In the anomalous behaviour recognition framework presented in this paper, the ultimate categorisation choice arises from the collaboration of numerous subtasks. A singular loss function, such as Binary Cross Entropy (BCE), while offering fundamental categorisation guidance, fails to enforce nuanced constraints on critical components (e.g., memory banks) inside the model. This paper develops a multi-objective composite loss function aimed at converting domain-specific prior knowledge (such as feature distinctions between normal and abnormal) and the structural advantages of the model (such as the memory bank) into precise, optimisable mathematical constraints that facilitate more efficient and robust learning for the model.

The design initiates with a principal classification objective. We utilise binary cross-entropy loss (BCE) as the principal supervisory term, which serves as the key source of gradients for comprehensive end-to-end training of the model. We employ the output of the attention mechanism, att, to calculate the anomaly score by averaging the top-k clips with the highest attention scores, thereby representing the anomaly probability of the entire video and effectively concentrating on critical moments while disregarding background noise. The Anomaly Loss of this core is delineated as follows:

(6) $$L_{an}=L_{BCE}\left(mean(topk(att)),label\right),$$

To maximise the utility of the normal and anomalous memory pools in the model, we implement a series of prototype memory restrictions aimed at establishing a “compact within-class, separated between-class” memory feature structure. The objective is to develop a memory feature structure that is “intra-class compact and inter-class separated.” To attain intra-class compactness, we compel the responses of abnormal samples in abnormal memory banks ($A_{att,}$) and normal samples in normal memory banks ($N_{att}$) to converge to 1 via the loss functions for abnormal samples, $L_{A}$, and normal samples, $L_{N}$, respectively: $L_{A}=L_{BCE}\left(A_{att},1\right)$ and $L_{N}=L_{BCE}\left(N_{att},1\right)$. To attain inter-class separation, we propose a “crossover” loss $L_{A-N}$ that compels the response of abnormal samples within the normal pool $A_{Natt}$ to approach 0. Additionally, we establish a more robust constraint, the abnormal loss of normal samples $L_{pan}$, to penalise elevated score responses of normal samples within the abnormal pool:

(7) $$L_{A-N}=L_{BCE}\left(A_{Natt},0\right),L_{pan}=L_{BCE}\left(mean(topk(1-N_{Aatt})),1\right)$$

Alongside enhancing the overall configuration of the feature space, we present general structural regularity terms that enforce geometric and distributional restrictions over the entire feature space. We present Triplet Loss, designed to enable the learnt features to establish an effective geometric structure in the metric space, positioning comparable samples in proximity and dissimilar samples at a distance. Simultaneously, we impose distributional and distance constraints: the KL Scatter Loss $L_{KL}$ is employed to avert the variance collapse of the latent Gaussian distribution model devised for normal data, thereby ensuring the smoothness of the latent space. At the same time, the Distance Loss ($L_{dis}$) directly enhances the separability of the final features by explicitly penalising the distances between normal and outlier features.

The comprehensive loss function is formulated as a weighted summation of the following components:

(8) $$L_{total}=L_{an}+\lambda_{1}\left(L_{A}+L_{pan}+L_{N}+L_{A-N}\right)+\lambda_{2}L_{tri}+\lambda_{3}L_{KL}+\lambda_{4}L_{dis},$$

The weight of the core classification loss, $L_{an}$, is set at 1.0, serving as the standard for optimisation. All remaining loss terms are auxiliary or regularisation components, with weights $\lambda_{i}$ that adjust the contributions of various learning objectives. For instance, $\lambda_{1}$ governs the level of oversight on the memory bank module, whereas $\lambda_{2}$, $\lambda_{3}$, and $\lambda_{4}$ modulate the structural limitations on the feature space and data distribution. These weights seek to establish an equilibrium that stabilises the training process and optimises the performance of the model.

## 4. Experiments

This section intends to assess our suggested method via a series of systematic experiments. We will evaluate the performance against contemporary state-of-the-art methodologies using the public datasets UCF-Crime [3] and XD-Violence [27]. Our experimental design will focus intently on the fundamental challenges addressed in the preceding section to systematically evaluate our proposed technical solutions. Firstly, we will assess the foundational timing modelling capabilities by juxtaposing its performance with that of conventional models, and evaluate whether the HMTE module can proficiently capture both long- and short-term interdependence of intricate events. We will determine the background suppression capability of the model via directional tests and performance comparisons in highly disturbed environments, and evaluate the anomalous prototype fitting ability of the bi-directional memory bank based on its performance with the label-ambiguous XD-Violence dataset. Ultimately, we will comprehensively validate the final feature discrimination capability of the model through specialised fine-grained anomaly classification tests to assess its efficacy in differentiating comparable, ambiguous abnormalities. This section seeks to illustrate that each design in our problem-driven validation approach provides a distinct technological solution, hence highlighting its specific advantages over current methodologies.

### 4.1. Dataset and Evaluation Criteria

To assess the efficacy of the proposed strategy, we performed comprehensive evaluations on two prominent anomaly detection datasets, UCF-Crime [3] and XD-Violence [27].

The UCF-Crime dataset comprises 13 categories of genuine anomalous incidents, using films extracted from unprocessed surveillance recordings. The training dataset includes 800 normal videos and 810 anomalous videos, whilst the test dataset consists of 150 normal videos and 140 anomalous videos. The primary problem with this dataset is that the abnormal signals are feeble. Simultaneously, the backdrop dynamics are pronounced: surveillance film frequently includes substantial dynamic background elements (e.g., pedestrians, traffic) that are irrelevant to the event, and numerous anomalous behaviours are visually indistinct from the periphery of normal behaviour. This presents a definitive technical validation case for our dual-stream attention method. In low signal-to-noise situations, the model must concentrate on critical spatial and temporal areas while efficiently attenuating background noise. Our attention module is the ideal solution for this, capable of identifying and extracting the most informative and suspicious portions from intricate situations through autonomous learning, hence validating its background suppression capability.

XD-Violence is an extensive and heterogeneous dataset of 4754 unedited videos sourced from web content, live sports broadcasts, and surveillance footage. The primary challenge with this dataset is the morphological diversity of the anomalies and the ambiguity of video-level labelling; specifically, we only ascertain that a lengthy film has anomalies without being able to determine their precise timing. This attribute underscores the necessity and benefit of our memory module. The model must learn a stable and generalisable collection of prototype features despite inaccurate labels and highly varied input patterns. Our learnable bi-directional memory module is intended for this objective: by storing and updating typical normal and abnormal patterns, it facilitates stable and reliable feature matching in extremely diverse data, therefore directly proving its capacity to accommodate atypical prototypes.

For the experimental evaluation, consistent with previous studies, we measure the performance of WSVAD using the area under the receiver operating characteristic curve (AUC) at the frame level on the UCF-Crime dataset. Meanwhile, we use the area under the precision–recall curve (AP) at the frame level for the XD-Violence dataset as the evaluation metric. Higher AUC and AP values indicate better network performance.

### 4.2. Experimental Details

We extract snippet features from the I3D [28] model pretrained on the Kinetics-400 dataset. The configuration for both datasets remains consistent. During training, a multi-crop aggregation method is employed to obtain the final anomaly scores, setting the number of crops for UCF-Crime to 10 and for XD-Violence to 5. To ensure robust performance when processing videos with extreme durations (tens of minutes to hours), our framework adopts a hierarchical temporal sampling strategy to manage computational complexity while maintaining anomaly localization accuracy effectively. Specifically, for videos exceeding 10 min, we first apply uniform frame down-sampling to reduce temporal redundancy while preserving essential motion and appearance cues. Next, we divide the entire video into overlapping temporal segments of fixed length, which are processed independently by the feature extractor and memory module. This segmentation enables our model to capture local spatio-temporal dynamics without being affected by the excessive length of the video. Finally, we aggregate the segment-level anomaly score by aligning the predicted scores to the original timeline. This hierarchical design ensures that the model remains computationally efficient and minimises the risk of anomaly localization loss in long-duration videos.

**Figure 5:**
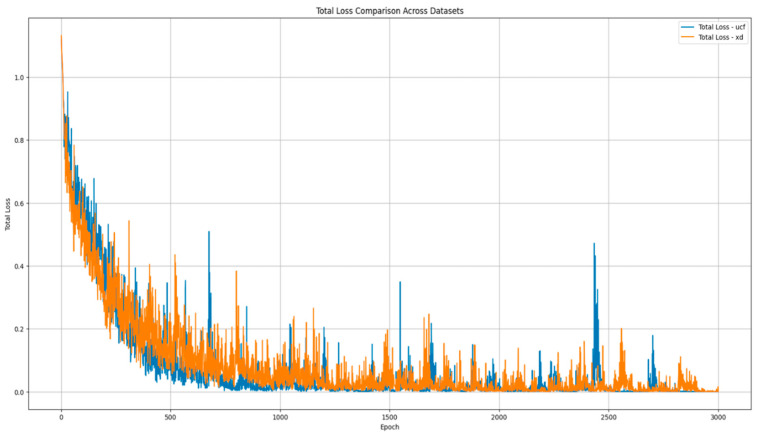
The change in training loss value over training time.

The optimal values for key hyperparameters were determined via a systematic grid search on a held-out validation set. The model is trained with a learning rate of 0.0001 and a batch size of 64 over 3000 iterations, with $M_{a}=70$ and $M_{n}=70$ for both UCF-Crime and XD-Violence. The hyperparameters ($\lambda_{1}$,$\lambda_{2},\lambda_{3}$,$\lambda_{4}$) are set to (0.1, 0.1, 0.001, 0.0001) to ensure a balanced total loss after 3000 training iterations for each dataset. The loss value of the model tends to stabilise, as illustrated in Figure 5, and the optimizer used is Adam. Our experiments were conducted on a machine equipped with an Intel (R) Xeon (R) Gold 6430 CPU (Intel Corporation, Santa Clara, CA, USA) and NVIDIA GeForce RTX 4090 GPU (NVIDIA Corporation, Santa Clara, CA, USA), using CUDA 12.1, Python 3.9.21, and Pytorch 2.1.0.

### 4.3. Experimental Results

#### 4.3.1. Performance Comparison with Competing Approaches on UCF-Crime

In the UCF-Crime dataset, we compared the AUC score of our method with the current mainstream VAD method, as shown in Table 1. Our model achieves an AUC score of 87.43%, and this superior performance reflects how well our model structure matches the challenges of this dataset. Unlike methods such as BN-WVAD [15] that simply optimise the network structure, our model not only optimises the network but also innovates at the level of feature representation and matching. This proves the feature discrimination capability of our method: learning the discriminative normal and abnormal prototypes through bi-directional memory units provides a powerful discriminative prior for the model. Moreover, in contrast to techniques such as UR-DMU [26] that also use memory networks, UR-DMU lacks feature purification before querying the memory bank. Our core innovation, the “Attention Before Memory” mechanism, utilises prototype knowledge more efficiently by using dual-stream attention as an intelligent filter to denoise and focus features before querying. This directly reflects the superior background suppression capability of the model.

#### 4.3.2. Performance Comparison with Competing Approaches on XD-Violence

The XD-Violence dataset necessitates that the model exhibits excellent generalisability across various situations and resilience to inaccurate labels. Table 2 demonstrates that our strategy attains an AP score of 85.51%. This accomplishment is primarily ascribed to the strength of our bidirectional memory bank. Our algorithm can simultaneously learn to identify “normal” and “abnormal” patterns from very varied and imprecisely labelled inputs, establishing persistent prototype libraries for both categories. This dual modelling capacity allows the model to render more generalised assessments when encountering movies from unfamiliar scenes. This unequivocally confirms the robust capacity of our model to accommodate atypical prototypes.

#### 4.3.3. Computational Efficiency

To evaluate the practical applicability of our proposed method, we conducted an analysis of its computational efficiency. We compared the parameter count and inference time of our model against several state-of-the-art methods, with the results presented in Table 3.

In terms of model size, our method is significantly more efficient, requiring only 0.01G parameters. This is half the size of RTFM [13] and an order of magnitude smaller than VadCLIP [33]. Regarding inference speed, our model demonstrates a substantial advantage, with a test time of just 0.03 s. This is over 4.5 times faster than the next fastest method, RTFM [13]. This analysis clearly demonstrates that our proposed model not only achieves high accuracy but also offers superior computational efficiency. Its low parameter count and fast inference speed make it highly suitable for real-world deployment scenarios where computational resources may be limited.

#### 4.3.4. Analysis of FAR and Background Suppression Capability

This section conducts a focused evaluation of the discriminative capability of our model, by analysing two critical dimensions: background suppression efficacy via false alarm rate assessment and feature discrimination proficiency through detailed classification analysis.

Firstly, to evaluate the background suppression efficacy of the model, we employ two complementary methodologies: frame-by-frame and window-based analyses, to thoroughly assess its false alarm rate (FAR), with results presented in Table 4. In the traditional frame-by-frame evaluation, the FAR of our model is only 6.98%, which is much lower than the comparison method. Moreover, in the windowed review, which more accurately reflects real-world applications, the FAR of our model is significantly diminished from 6.98% to 5.21%. In contrast, the FAR of the comparison approach increases slightly instead. This comparison clearly illustrates that the erroneous alerts produced by our model primarily result from decentralised transient noise, which can be efficiently mitigated using temporal smoothing. This outcome offers substantial evidence for both the enhanced accuracy and the robust background suppression ability of the model.

This paper further breaks down the primary error sources contributing to the FAR to provide a deeper insight into the failure modes of the model. We manually analysed and categorised the top 100 false alarm instances from the UCF-Crime dataset, with the results summarised in Table 5. The analysis reveals that the majority of false alarms are caused by sudden illumination changes (41%), followed by complex background object motion (34%) and activities in crowded scenes (25%). This finding suggests that while our model is generally robust, future optimizations could focus on improving invariance to these challenging scenarios.

**Figure 6:**
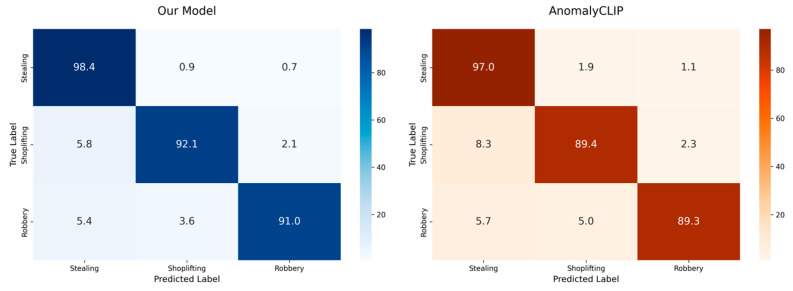
Fine-grained anomaly discrimination performance.

Secondly, a robust model must not only suppress the background but also accurately differentiate semantically comparable anomalous events. To validate the advantage of our model in feature discrimination capability, we compared it against the AnomalyCLIP model [4], which also possesses fine-grained recognition abilities. We conducted a fine-grained classification experiment, with the results illustrated in a confusion matrix in Figure 6.

To allow for a clearer quantitative assessment of where our prototype learning excels or fails, we calculated the per-class Precision, Recall, and F1-Score based on the confusion matrix. These results are presented in Table 6. The quantitative metrics reveal that our model achieves an exceptionally high recall of 98.4% for the ‘Stealing’ class, indicating its learned prototypes are highly effective at identifying nearly all instances of this event. While its precision for ‘Stealing’ (89.8%) is impacted by some confusion with the visually similar ‘Shoplifting’ and ‘Robbery’ classes, the model demonstrates high precision for ‘Robbery’ (97.0%) and a strong balance for ‘Shoplifting’. This detailed analysis provides a deeper insight into the specific strengths and trade-offs of our prototype learning approach.

Figure 6 illustrates that the prediction outcomes of our model are predominantly aligned around the diagonal line when addressing the highly ambiguous categories such as “theft,” “shoplifting,” and “robbery,” whilst the non-diagonal values of the comparative model are also significantly concentrated along the diagonal line. The off-diagonal values of the model are markedly elevated, signifying considerable category confusion. This provides clear proof that our bi-directional memory bank can learn more distinct prototypes for various but analogous anomalous events, leading to more accurate decision boundaries in the feature space.

By synthesising these results, we can directly attribute the enhancement in performance to the structural improvements of the model. The efficacy of UCF-Crime arises from the concentration capabilities of the Attention mechanism and the temporal modelling proficiency of HMTE, which collectively improve background suppression. The efficacy of XD-Violence derives from the implementation of the Memory mechanism, which creates durable prototypes and enhances the capacity for anomalous prototype fitting. This “attention focusing, then memory matching” approach is likely to possess strong generalisability. Even with other datasets exhibiting greater incompleteness in modalities or labels, its fundamental mechanism of dynamically filtering information via attention and stabilising prototypes through memory bank learning will uphold performance stability. It may be regarded as a significant structural contribution.

### 4.4. Parameter Sensitivity Analysis

The dual memory unit based on bidirectional spatial attention mechanism achieves optimal performance by simultaneously storing two types of memory patterns. To investigate the appropriate number of memory libraries, we set up nine memory cases to seek the optimal configuration for the number of memory modules. The results are shown in Table 7. Here, $M_{a}\;$ and $M_{n}$ represent the number of anomalous and normal memories, respectively. When $M_{a}=70$ and $M_{n}=70$, our method achieved optimal performance on both datasets.

### 4.5. Ablation Study

To systematically assess the efficacy of each core component in the model and to distinctly identify performance improvements attributable to a specific mechanism rather than mere structural complexity, we executed a series of ablation experiments adhering closely to the modular design framework. Commencing with a baseline model (Baseline) that comprises solely the foundational backbone network, we sequentially incorporate HMTE, PGRN, DFRL, and DS-AEMN modules, monitoring the performance variations to establish a definitive causal relationship. The experimental findings are presented in Table 8.

Analysis of individual values for baseline and singular modules: The baseline model attained an AUC of 86.26% and an AP of 82.80% on UCF-Crime and XD-Violence, respectively. Consequently, we assessed the distinct worth of each module. Upon the sole introduction of the HMTE module, performance enhances to 86.37% and 84.00%, illustrating the autonomous and prompt advantage of capturing local timing dependencies through temporal convolutional networks. The introduction of the DFRL module as a standalone support structure also led to enhanced performance, validating the efficacy of residual connection in stabilising training and facilitating the acquisition of robust features. The incorporation of the core DS-AEMN module yields the most substantial single-module performance enhancements of 86.60% and 84.35%, thereby affirming that our fundamental principle of “notice first, remember later” is an exceptionally effective strategy for differentiating ambiguous anomalous events, exhibiting robust independent discriminative capability. The basic principle of “attention first, then memory” is an effective method for identifying ambiguous abnormal events, demonstrating significant independent discriminatory capability.

Examination of the synergistic impact of multi-module integration: Upon confirming the independent validity of each module, we further investigated their synergistic effect. The integration of HMTE and DFRL elevates the AUC on UCF-Crime to 86.99%, demonstrating that the superior feature backbone and improved temporal modelling capacity collectively establish a more robust feature foundation for subsequent processing. The fundamental DS-AEMN module provides substantial performance improvements when integrated with other modules. The integration of Base + HMTE + DS-AEMN attained an 85.30% average precision on XD-Violence. This illustrates the synergistic relationship between the modules: with access to a practical temporal context (provided by HMTE), our DS-AEMN module can execute attentional focusing and memory matching with greater precision, demonstrating that its efficacy is enhanced not only in isolation but also through the collaborative framework.

The comprehensive model, incorporating all three modules, attained optimal performance with an AUC of 87.43% and an AP of 85.51%. To specifically isolate the contribution of the PGRN, we conducted an additional experiment where only the PGRN module was removed from the full model. As shown in Table 8, this led to a notable performance drop to 87.18% AUC on UCF-Crime and 85.12% AP on XD-Violence. This result quantitatively confirms the significant impact of PGRN in capturing long-range, content-driven global dependencies, which complements the local features from HMTE. This outcome illustrates the synergistic interaction among the modules: the HMTE captures the temporal context, the PGRN establishes global relations, the DFRL ensures the depth and stability of feature extraction, and the DS-AEMN effectively identifies anomalies through an accurate prototype matching task within this framework. Each component addresses the difficulty from a distinct perspective, and their combination becomes a synergistic partnership rather than a mere performance overlay, culminating in enhanced model performance.

To further verify the contribution of each component within our dual-stream attention mechanism, we performed a fine-grained ablation study, as shown in Table 9. The results indicate that incorporating either the spatial or the channel attention branch individually yields a performance gain over the baseline, confirming the effectiveness of each component. Critically, the synergistic use of both branches achieves the best performance on both datasets. This strongly demonstrates that our dual-stream design is more than a simple stacking of components; it effectively captures complementary ‘where’ (spatial) and ‘what’ (channel) information to achieve efficient feature purification, thereby validating the rationale and necessity of our approach.

The efficacy of the composite loss function (Equation (8)) is somewhat contingent upon the hyperparameters $\lambda_{i}$ associated with each loss weight. A systematic sensitivity analysis is conducted on both the UCF-Crime and XD-Violence datasets to demonstrate that the selected hyperparameters ($\lambda_{1}=0.1$, $\lambda_{2}=0.1$, $\lambda_{3}=0.001$, $\lambda_{4}=0.0001$) are not overfitting to a single dataset but represent a generalised choice that performs robustly across varying data characteristics. The tests utilised the control variable method, assessing one parameter while maintaining all other parameters at their final selected values.

**Figure 7:**
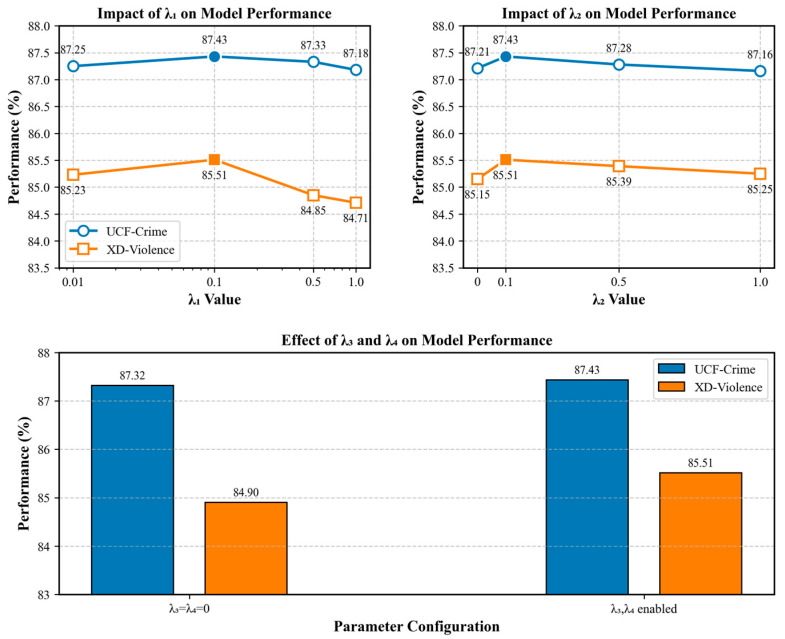
Impact of $\lambda_{i}$ on model performance.

Impact of memory loss weight $\lambda_{1}$: Figure 7 illustrates that the effect of $\lambda_{1}$ exhibits a very similar pattern across both datasets. The performance on both UCF-Crime and XD-Violence reaches its zenith at $\lambda_{1}=0.1$. When the weights are very low (0.01), the supervised limitations on the memory module are inadequate, resulting in suboptimal utilisation of its capacity. Simultaneously, when the weights exceed 0.5, the high auxiliary loss disrupts the primary classification job, resulting in a concurrent decline in performance across both datasets. The uniformity across datasets compellingly illustrates that $\lambda_{1}=0.1$ is a robust and optimal parameter, irrespective of data distribution.

Effect of the Triplet loss weight $\lambda_{2}$: Likewise, the examination of $\lambda_{2}$ demonstrates a comparable trend. Eliminating this factor ($\lambda_{2}=0$) results in a notable decline in performance across both datasets, thus affirming the critical necessity of explicitly enforcing the structure of the eigenspace metric to enhance model generalisation. Optimal performance is reliably achieved at $\lambda_{2}=0.1$, but increased weights detrimentally affect performance due to overregularization.

Combined impact of KL divergence and distance loss ($\lambda_{3},\lambda_{4}$): $\lambda_{3}$ and $\lambda_{4}\;$ function as more refined regularisation terms, which we jointly ablate. The table indicates that both datasets exhibit a minor decline in performance from the ideal position when both losses are simultaneously eliminated. Despite the lesser magnitude of the effect compared to $\lambda_{1}$ and $\lambda_{2}$, it suggests that these two regularisation terms, employed to stabilise the latent space and explicitly separate features, are generally advantageous for subsequent fine-tuning and enhancing model performance.

This series of ablation experiments over two distinct characterisation datasets establishes a robust empirical foundation for our ultimate hyperparameter design. The consistently reliable performance patterns in the results suggest that the selected weights are not mere “fortunate values” that overfit a particular data distribution, but relatively resilient selections that equilibrate the loss terms and enhance the generalisation capacity of the model.

### 4.6. Qualitative Results

To enhance the visualisation of the efficacy of our method and to substantiate the preceding quantitative analysis, we present the findings of our qualitative investigation on the UCF-Crime and XD-Violence datasets.

**Figure 8:**
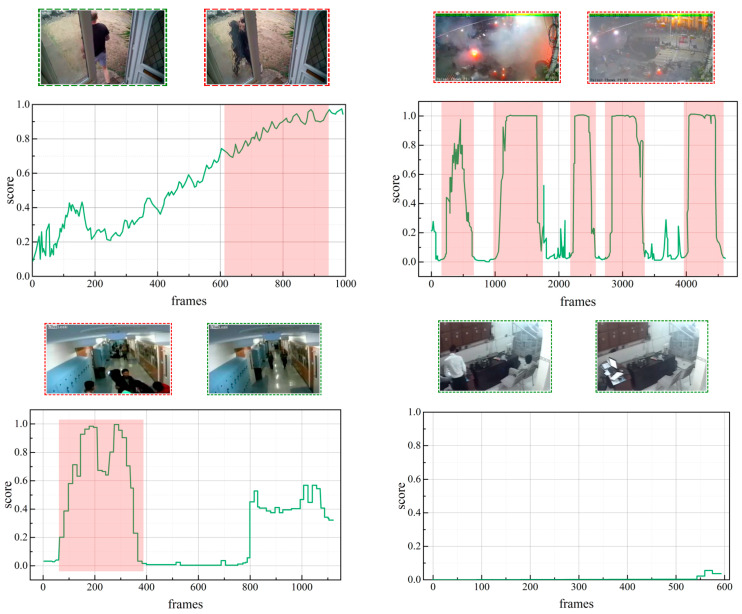
Visual analysis of the results of the proposed method on the UCF-Crime and XD-Violence datasets. The anomaly score of the model is plotted with a green curve. The light pink shaded regions denote ground-truth data for anomalous frames. Representative snippets of abnormal and benign events are displayed within red and green boxes for comparison.

The anomaly score curves depicted in Figure 8 provide a visual confirmation of the proficiency of our model in time-series modelling. Specifically, the figure provides qualitative evidence of the ability of the model to handle anomalies of varying durations. For instance, the model successfully localises both a long-duration event with a gradually rising score (left plot) and a series of brief, explosive anomalies with sharp, spiky scores (right plot). This demonstrates that our HMTE module, designed to capture both long- and short-term dependencies, is effective in practice. The overall alignment of the scores of the model with the actual abnormal periods (shown by the pink shaded areas) shows that, even in a poorly guided context without precise frame-level annotations, our approach can successfully acquire dependable spatio-temporal patterns and attain accurate anomaly localisation. A detailed quantitative analysis on this topic is a valuable direction for future work.

**Figure 9:**
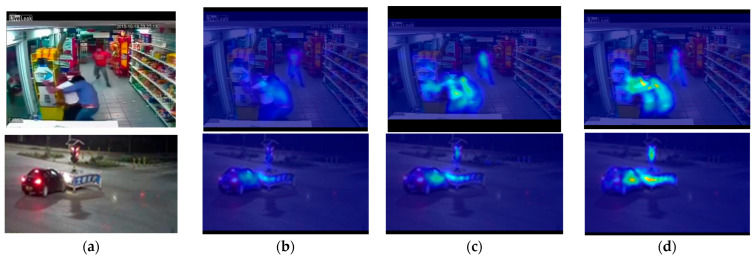
Illustration of the role of the dual-stream attention mechanism in detection via heatmaps. (**a**) Original video frame; (**b**) before adding attention mechanism; (**c**) adding channel attention; (**d**) adding channel and spatial dual-stream attention mechanism.

Figure 9 illustrates the distinct performance of this technique on Fighting028_x264 and Vandalism004_x264 within the UCF-Crime dataset, which offers the most explicit visual demonstration of the background suppression capacity of our model. The graphic illustrates that, following the implementation of the dual-stream attention mechanism (from c to d), the focus of the network progressively shifts towards the primary topics (e.g., the combatants) in the image, away from the dispersed background areas. This incremental purification approach successfully mitigates the influence of extraneous background information, hence augmenting the accuracy of the model in learning video features and ultimately enhancing overall recognition performance.

## 5. Conclusions

This study addresses key challenges in WSVAD, namely ambiguous anomaly characteristics, significant background interference, and the inadequate modelling of intricate temporal dependencies. To tackle these issues, a dual-stream attention and memory-enhanced network is proposed, transforming the conventional implicit feature learning approach into an innovative framework. This framework integrates multi-scale temporal modelling with explicit prototype discrimination to address the inherent uncertainty in weakly supervised settings systematically. At the temporal modelling level, an enhanced time-series encoder is developed by incorporating multi-scale dilated convolutions with residual connections, which effectively addresses feature degradation in deep networks and enhances the capacity to capture long- and short-term dependencies. More centrally, at the feature discrimination level, a dual-stream attention and memory-enhanced network is introduced. This design converts implicit feature learning into explicit prototype matching via independent memory for normal and abnormal prototypes, fundamentally addressing the issue of indistinct boundaries. Comprehensive studies on the UCF-Crime and XD-Violence datasets demonstrate that the proposed approach surpasses existing mainstream methods across multiple critical measures.

In conclusion, this research presents a high-performance VAD model and, more significantly, an effective paradigm of “attention focus and prototype matching” for resolving uncertainty in weakly supervised learning. Future work will advance in two directions: first, to enhance the cross-domain generalisation capability of the model, we will investigate adaptive, unsupervised updating mechanisms for memory prototypes to reduce reliance on pseudo-labels. The effectiveness of this new approach will be validated by extending our evaluation to additional datasets such as ShanghaiTech and Avenue. Second, while the current framework only utilises RGB inputs, developing a cross-modal attention method utilising multimodal information, such as optical flow or audio data, could improve the discriminative ability of the model in more intricate scenarios, particularly for events with weak visual cues.

## Figures and Tables

**Table 1:** Evaluating the effectiveness of different methods on UCF-Crime.

Supervision	Year	Method	Feature	AUC(%)
Unsupervised	2022	GCL [11]	ResNext	71.04
	2023	DyAnNet [10]	I3D-RGB	79.76
	2024	C2FPL [9]	I3D RGB	80.65
	2024	CLAP [12]	I3D RGB	80.90
Weakly supervised	2022	GCN [19]	C3D RGB	81.08
	2022	GCN [19]	TSN RGB	82.12
	2022	GCN [19]	TSN FLOW	78.08
	2023	MGFN [29]	I3D-RGB	86.98
	2023	MGFN [29]	VideoSwin-RGB	86.67
	2023	UR-DMU [26]	I3D-RGB	86.34 *
	2024	AnomalyCLIP [4]	ViT-B/16	86.36
	2024	BN-WVAD [15]	I3D-RGB	87.24
	2024	OE-CTST [18]	I3D-RGB	86.37
	2024	OE-CTST [18]	VideoSwin-RGB	86.92
	2024	MIL-BERT [23]	I3D-RGB + Flow	86.71
	2025	MTFL [20]	VST-RGB	87.16
	2025	ViCap-AD [30]	I3D-RGB	87.20
	-	Ours	I3D-RGB	87.43

* Indicates that the method is re-trained.

**Table 2:** Evaluating the effectiveness of different methods on XD-Violence.

Supervision	Year	Method	Feature	AP(%)
Semi-supervised	2016	Hasan et al. [31]	-	30.77
	2019	CoMo [7]	I3D-RGB	81.30
Weakly supervised	2021	RTFM [13]	C3D	77.81
	2023	MGFN [29]	VedioSwin-RGB	80.11
	2023	Cu-Net [17]	I3D-RGB	78.74
	2023	Cu-Net [17]	I3D-RGB+VGGish	81.43
	2023	CLIP-TSA [32]	Clip Vit	82.17
	2023	UR-DMU [26]	I3D-RGB	82.30 *
	2023	MGFN [29]	I3D-RGB	79.19
	2023	MGFN [29]	VideoSwin-RGB	80.11
	2024	AnomalyCLIP [4]	ViT-B/16	78.51
	2024	OE-CTST [18]	I3D-RGB	80.56
	2024	OE-CTST [18]	VideoSwin-RGB	81.78
	2024	BN-WVAD [15]	I3D-RGB	84.09 *
	2024	MIL-BERT [23]	I3D-RGB + Flow	82.10
	2025	MTFL [20]	VST-RGB	84.57
	2025	ViCap-AD [30]	I3D-RGB	85.02
	-	Ours	I3D-RGB	85.51

* Indicates that the method is re-trained.

**Table 3:** Computational efficiency comparison.

Method	Params (G)	Test Time (s)
RTFM [13]	0.02	0.14
MIST [16]	0.03	0.25
VadCLIP [33]	0.35	0.27
Ours	0.01	0.03

**Table 4:** False Alarm Rate (FAR) comparison under different evaluation protocols.

Method	FAR (Abnormal)	
	Original (%)	Window (%)
UR-DMU [26]	11.60	12.10
BN-WVAD [15]	21.40	21.67
Ours	6.98	5.21

**Table 5:** Breakdown of False Alarm Error Sources on UCF-Crime.

Error Source	Examples	False Alarm Proportion
Illumination Changes	Shadows, headlights, day-night transitions	41%
Background Object Motion	Moving trees, camera shake, passing cars	34%
Crowed Scenes	Group activities, dense human motions	25%
Total	100 analysed false alarms	100%

**Table 6:** Per-class fine-grained performance.

Anomaly Class	Precision (%)	Recall (%)	F1-Score (%)
Stealing	89.8	98.4	93.9
Shoplifting	95.3	92.1	93.7
Robbery	97.0	91.0	93.9

**Table 7:** Comparison of the results for $M_{a}$ and $M_{n}$ with different amounts of memory.

($M_{a},\;M_{n}$)	DataSet			
	UCF (%)		XD (%)	
	AUC	AP	AUC	AP
(20, 20)	87.35	35.32	93.77	83.48
(30, 30)	86.13	30.02	93.44	82.84
(40, 40)	85.56	29.30	93.31	82.85
(50, 50)	85.95	30.82	93.74	84.15
(60, 60)	86.60	33.84	93.70	84.70
(70, 70)	87.43	35.67	93.82	85.51
(80, 80)	85.38	31.29	93.03	83.32
(90, 90)	86.10	31.75	93.63	83.00
(100, 100)	85.64	31.75	94.26	84.30

**Table 8:** Comparison of ablation results. ✓ and ✗ represent our backbone network with or without specific modules, respectively.

Modules				Results	
HMTE	PGRN	DFRL	DS-AEMN	UCF AUC (%)	XD AP (%)
✗	✗	✗	✗	86.26	82.80
✓	✓	✗	✗	86.37	84.00
✗	✗	✓	✗	86.33	83.98
✗	✗	✗	✓	86.60	84.35
✓	✓	✓	✗	86.99	84.95
✓	✓	✗	✓	87.35	85.30
✗	✗	✓	✓	86.69	84.70
✓	✗	✓	✓	87.18	85.12
✓	✓	✓	✓	87.43	85.51

**Table 9:** Ablation study on the dual-stream attention mechanism. ✓ and ✗ denote the model with or without the corresponding attention branch.

Spatial Attention	Channel Attention	UCF AUC (%)	XD AP (%)
✗	✗	86.79	84.65
✓	✗	87.23	85.26
✗	✓	87.14	85.15
✓	✓	87.43	85.51

## Data Availability

The original contributions presented in this study are included in the article. Further inquiries can be directed to the corresponding author.
